# PAIDB v2.0: exploration and analysis of pathogenicity and resistance islands

**DOI:** 10.1093/nar/gku985

**Published:** 2014-10-21

**Authors:** Sung Ho Yoon, Young-Kyu Park, Jihyun F. Kim

**Affiliations:** 1 Synthetic Biology and Bioengineering Research Center, Korea Research Institute of Bioscience and Biotechnology (KRIBB), Daejeon 305-806, Republic of Korea; 2 Biosystems and Bioengineering Program, Korea University of Science and Technology, Daejeon 305-350, Republic of Korea; 3 Bio-Medical Science Co., Ltd., Daejeon 305-301, Republic of Korea; 4 Department of Systems Biology, Yonsei University, Seoul 120-749, Republic of Korea

## Abstract

Pathogenicity is a complex multifactorial process confounded by the concerted activity of genetic regions associated with virulence and/or resistance determinants. Pathogenicity islands (PAIs) and resistance islands (REIs) are key to the evolution of pathogens and appear to play complimentary roles in the process of bacterial infection. While PAIs promote disease development, REIs give a fitness advantage to the host against multiple antimicrobial agents. The Pathogenicity Island Database (PAIDB, http://www.paidb.re.kr) has been the only database dedicated to providing comprehensive information on all reported PAIs and candidate PAIs in prokaryotic genomes. In this study, we present PAIDB v2.0, whose functionality is extended to incorporate REIs. PAIDB v2.0 contains 223 types of PAIs with 1331 accessions, and 88 types of REIs with 108 accessions. With an improved detection scheme, 2673 prokaryotic genomes were analyzed to locate candidate PAIs and REIs. With additional quantitative and qualitative advancements in database content and detection accuracy, PAIDB will continue to facilitate pathogenomic studies of both pathogenic and non-pathogenic organisms.

## INTRODUCTION

Increased awareness of infectious diseases of humans, animals and plants caused by microbial pathogens has accelerated the genome-wide study of microbial pathogenicity, called pathogenomics (1–3). Genomic islands (GIs) are regions of the genome that are acquired through horizontal gene transfer (HGT) (4). The genomes of pathogenic bacteria often contain pathogenicity islands (PAIs), a subset of GIs that mediate the horizontal transfer of genes encoding numerous virulence factors. Some known PAIs include the type III secretion system (e.g. LEE PAI in pathogenic *Escherichia coli* and Hrp PAI in *Pseudomonas syringae*), superantigen (e.g. SaPI1 and SaPI2 in *Staphylococcus aureus*), colonization factor (e.g. VPI in *Vibrio cholerae*), iron uptake system (e.g. SHI-2 in *Shigella flexneri*) and enterotoxin (e.g. *espC* PAI in *E. coli* and *she* PAI in *S. flexneri*). PAIs confer virulence upon the recipient, resulting in the dissemination and diversification of bacterial pathogens (5).

Antimicrobial resistance islands (REIs) are another class of GIs that are linked to pathogenesis by conferring simultaneous resistance to multiple antibiotics and facilitating the emergence of multidrug-resistant pathogens (6–8). For example, acquisition of the staphylococcal cassette chromosome *mec* (SCC*mec*) resulted in the emergence of methicillin-resistant *S. aureus* (9). The *Salmonella* genomic island 1 (SGI1) is associated with the multiple-drug-resistant form of *Salmonella typhimurium* (10). *Pseudomonas aeruginosa* genomic island 1 (PAGI-1) is found in the majority of clinical isolates (11). AbaR1 was reported to contain over 85% of resistance genes of *Acinetobacter baumannii* AYE, explaining a remarkable ability of this emerging opportunistic pathogen to rapidly acquire multidrug resistance within a few decades (12).

Pathogenomic studies necessitate specialized data resources related to pathogens. Public database servers have been developed for searching virulence factors (e.g. VFDB (13), MvirDB (14)) and PAIs (e.g. PAIDB (15), PAI-IDA (16), PredictBias (17), IslandViewer (18)). A recently developed software suite, PIPS (19), was specifically designed to predict PAIs, but requires installation of multiple programs and databases on a Linux computer. Compared with most PAI-related databases, which focus on predicting PAIs by searching for HGT (20), PAIDB remains the only database dedicated to providing comprehensive information on all annotated and predicted PAIs in prokaryotic genomes (21). PAIDB also allows users to predict PAI-like regions that are homologous to known PAIs using an automated identification system. Several databases of resistance genes have also been described, such as ARDB (22), CARD (23) and BacMet (24). Although numerous REIs have been reported, to our knowledge, a REI-related database has yet to be developed.

In 2007, we released PAIDB, which contained 112 types of PAIs and 889 GenBank accessions of complete or partial PAI loci previously described in 497 pathogenic bacterial strains (15). Since the release of PAIDB, there have been continuous requests for an expanded collection of PAIs and candidate regions in newly sequenced genomes (21). Here, we demonstrate PAIDB v2.0, which contains 223 types of PAIs from 1331 accessions, and 88 types of REIs from 108 accessions. This update to the PAIDB reflects a dramatic increase in the number of analyzed genomes, improved accuracy of candidate region detection and a functional update of the web application.

## DATABASE CONTENT EXPANSION

### Definition of terms

**Figure 1.**
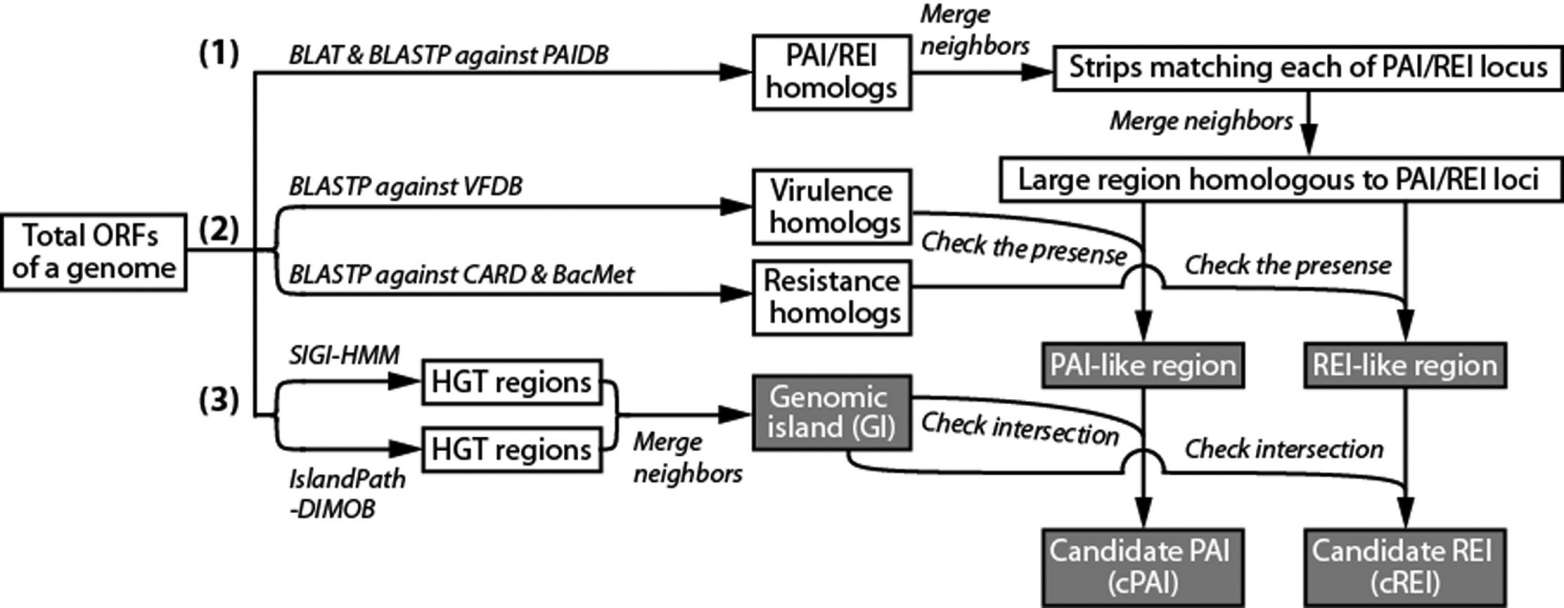
Procedure for identifying candidate PAIs and REIs in a sequenced genome. The DNA and amino acid sequences of a genome are processed as follows. (1) Genomic regions homologous to PAI and REI loci are identified by BLAT and BLAST searches against PAIDB. (2) The existence of known virulence and resistance genes in a genomic region is checked through BLASTP searches against VFDB, CARD and BacMet databases. (3) The PAI-like region is examined for overlapping GIs through detection of HGT regions.

We have previously defined a ‘PAI-like region’ as a predicted genomic region that is homologous to known PAI(s) and contains at least one virulence gene homolog from the PAI loci (15,25). If a PAI-like region overlaps a GI, we call it a ‘candidate PAI (cPAI)’, otherwise the region is a ‘non-probable PAI (nPAI)’. Likewise, in this study, a REI-like region overlapping GI(s) was dubbed as a cREI and a REI-like region not overlapping a GI as an nREI (Figure 1).

### PAI and REI data

GenBank accession numbers for PAI and REI loci were collected via an exhaustive search of GenBank and academic literature using a variety of terms related to ‘pathogenicity island’ and ‘resistance island’ (Supplementary Table S1). We also added PAIs and REIs that were reported in genome sequencing papers in a GenBank-like flat file format (Supplementary Table S2). Via expert review, we collected 223 types of PAIs, consisting of 1331 accessions for complete or partial PAI loci previously described in 804 pathogenic bacterial strains. Similarly, we collected 88 types of REIs with 108 accessions from 99 bacterial strains (Table 1).

**Table 1.** Statistics of PAI and REI loci that were collected through literature search (see Supplementary Tables S1 and S2 for the complete list of collected PAI and REI loci.)

Pathogen (number of strains)^a^	PAI			REI		
	Type	Accn^b^	ORF	Type	Accn^b^	ORF
*Acinetobacter baumannii* (38)	0	0	0	36	39	1024
*Aliivibrio salmonicida* (1)	0	0	0	1	1	36
*Bacteroides fragilis* (2)	1	2	10	0	0	0
*Bartonella tribocorum* (2)	4	4	104	0	0	0
*Burkholderia cenocepacia* (1)	0	0	0	1	1	58
*Campylobacter coli* (1)	0	0	0	1	1	15
*Citrobacter* sp. (2)	2	2	43	0	0	0
*Clavibacter michiganensis* (1)	1	1	90	0	0	0
*Clostridium* sp. (2)	7	7	62	0	0	0
*Corynebacterium* sp. (13)	21	39	940	6	9	209
*Cronobacter sakazakii* (1)	2	2	141	0	0	0
*Dichelobacter nodosus* (1)	2	4	57	0	0	0
*Enterobacter cloacae* (1)	1	1	1	0	0	0
*Enterococcus* sp. (8)	3	10	292	2	2	113
*Erwinia amylovora* (1)	1	8	93	0	0	0
*Escherichia coli* (142)	34	212	2517	2	2	73
*Francisella* sp. (9)	2	12	179	0	0	0
*Helicobacter* sp. (407)	2	618	1384	0	0	0
*Klebsiella pneumoniae* (6)	3	5	35	1	1	56
*Listeria* sp. (5)	4	24	151	0	0	0
*Lysinibacillus sphaericus* (1)	2	2	25	0	0	0
*Neisseria* sp. (14)	9	18	204	0	0	0
*Pasteurella multocida* (1)	0	0	0	1	1	96
*Photorhabdus luminescens* (1)	5	5	191	0	0	0
*Porphyromonas gingivalis* (1)	1	1	5	0	0	0
*Proteus mirabilis* (8)	1	1	97	1	7	494
*Pseudomonas* sp. (40)	19	55	1395	5	6	317
*Rhodococcus equi* (1)	1	1	9	0	0	0
*Salmonella* sp. (51)	28	84	1343	2	2	70
*Shigella* sp. (11)	5	15	252	1	1	70
*Sodalis glossinidius* (1)	2	2	61	0	0	0
*Staphylococcus* sp. (39)	24	67	2298	27	34	1393
*Streptococcus* sp. (10)	14	16	664	0	0	0
*Streptomyces turgidiscabies* (1)	1	5	34	0	0	0
*Vibrio* sp. (38)	8	69	541	1	1	2
*Xanthomonas* sp. (9)	4	11	255	0	0	0
*Yersinia* sp. (14)	9	28	467	0	0	0
Total (885 ea)	223	1331	13940	88	108	4026

^a^Number of strains that belong to the genus.

^b^GenBank accession or loci collected from genome sequences of pathogens

### Potential PAIs and REIs in prokaryotic genomes

**Figure 2.**
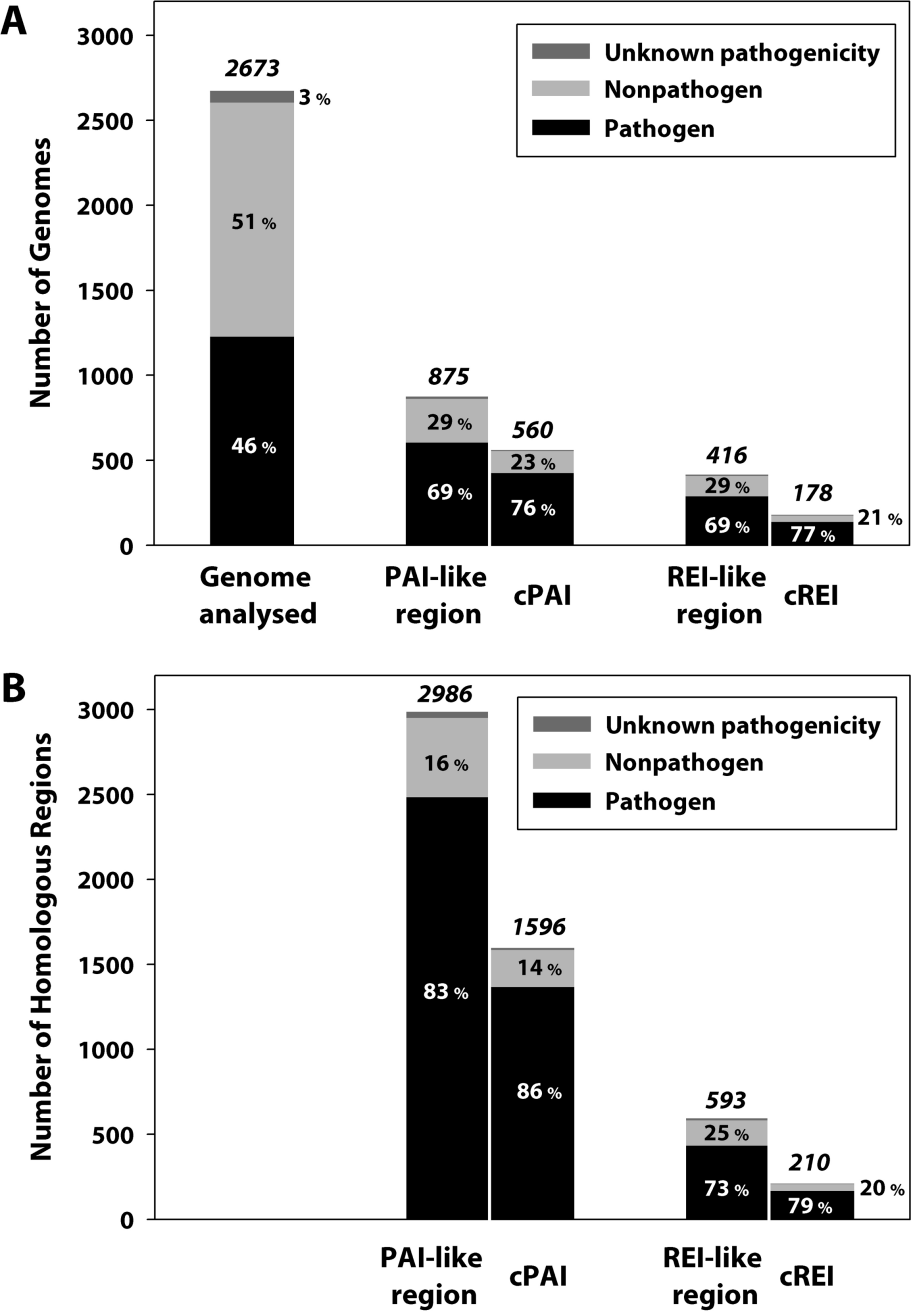
Number distribution of genomic regions homologous to the reported PAIs and REIs in 2673 prokaryotic genomes. (**A**) Barplot of numbers of genomes containing at least one homologous region. (**B**) Barplot of numbers of homologous regions. In each stacked bar, the total number is denoted on the top and the proportion (as a percentage) is shown inside, according to the organism's pathogenicity status—pathogenic (black), non-pathogenic (light gray) and unknown pathogenicity (dark gray). In a group of barplots for predicted regions, the left bar denotes the total number related to homologous regions, and the right bar represents the number related to candidate regions.

As of October 2013, the sequence files of 2673 prokaryotic genomes (including 160 archaea) had been downloaded from the NCBI FTP server (Supplementary Table S3). To determine the pathogenicity of the retrieved organisms, we referred to related publications and to the Genomes Online Database (GOLD) (26). We considered an organism pathogenic if any of the bacterial strains caused any adverse effects in any host—human, animal, bird, fish, insect or bacteria. Aside from the 70 organisms without pathogenicity information, we tagged 1226 organisms as pathogenic and 1377 as non-pathogenic (Supplementary Table S3). The genomes were analyzed to predict potential PAIs and REIs, producing 3579 regions that were PAI-like or REI-like in 966 strains. Of these regions, 1596 cPAIs were detected in 560 strains and 210 cREIs were found in 178 strains (Figure 2, Supplementary Table S4). In total, 49.3% of the pathogenic strains (604 ea) were predicted to have 1366 cPAIs. Intriguingly, 424 cPAIs were also found in 18.6% of the non-pathogenic genomes (256 ea). In contrast to cPAIs, cREIs were detected in a relatively small number of genomes (137 pathogenic and 38 non-pathogenic).

## METHODOLOGIES IMPROVEMENT

**Figure 3.**
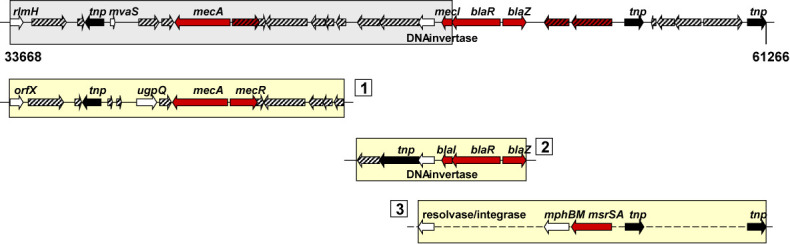
Example of detection of a candidate REI in a genome sequence. A 27.6 kb genomic region in the chromosome of methicillin-resistant *S. aureus* ST80-IV (GenBank accession number: NC_017351) was identified as a cREI by merging genomic regions homologous to known REI loci (yellow bar). The stitched together genomic region contains homologs of seven resistance genes from REI loci and CARD datasets (red arrow). The region spans a GI (gray bar) and has a G+C content (-2.56%, *P*-value ≈ 0) lower than that of the rest of the chromosome. Therefore, this REI-like region is considered as a cREI. Red arrows in yellow bars denote resistance genes. Transposase genes are colored black and hypothetical genes are hatched. Twenty-five reported REIs are homologous to this region, and three of them are shown: 1. SCC*mec* (GenBank accession number: AB033763, host: *S. aureus* NCTC10442); 2. ΦSh1 (PAIDB accession number: NC_007168_R2, *S. haemolyticus* JCSC1435); 3. πSh1 (PAIDB accession number: NC_007168_R3, *S. haemolyticus* JCSC1435; note that the order of genes in this locus is different from that of matched genes). Details can be explored on the PAIDB website (http://www.paidb.re.kr).

To detect candidate regions in genome sequences, we modified the method previously described in (25) (Figure 1). In a given genome sequence, each open reading frame (ORF) was searched for homology against the collected PAI and REI dataset at the nucleotide and amino acid level using BLAT (27) and BLAST+ (28), respectively. If the identity of the resulting hit was over 80% for a DNA sequence of a non-protein coding ORF (e.g. tRNA, rRNA and pseudogene), or 40% for a protein sequence, and the aligned region was both over 70% of the length of the query and the hit, the pair of sequences was considered as a homolog. Overlapping or adjacent genomic regions corresponding to the same or different PAI and REI loci were joined into a larger region (Figure 3). Small genomic regions below 8 kb in size were excluded (20). Of these regions, PAI-like or REI-like regions were identified by checking for the presence of at least one virulence or resistance gene homolog, respectively. Finally, a region was considered as a cPAI or cREI only if the PAI-like or REI-like region partly or entirely spanned a GI. The remaining set of regions that did not span a GI was denoted as nPAIs or nREIs. We detail further updates in the methods for detecting GIs, virulence factors and resistance genes in the following sections.

### Detection of genomic islands

GIs are a heterogeneous class of mobile elements that contain a large collection of genes acquired by HGT. Various methods have been suggested for their detection in microbial genomes (20). In the original version of PAIDB (15), genes were considered as acquired by HGT if their G+C content and codon usage were both aberrant (25). By merging neighboring HGT genes, a GI was identified. However, the *P*-value for codon usage deviation was calculated assuming a normal distribution of codon frequencies, which was later suggested to be suboptimal (29). Hence, to detect HGT regions in this update we have used SIGI-HMM (30), which measures the codon adaptation index, and IslandPath-DIMOB (31), which uses dinucleotide bias in combination with the presence of mobility gene(s). Both methods were reported to be the most accurate methods for GI predictors (32) and were applied in the IslandViewer web server (18). HGT regions detected from these methods were merged into a larger GI as described previously (25).

### Identification of virulence and resistance genes in candidate regions

In our detection scheme, the presence of virulence- or resistance-related genes is a crucial criterion to identify candidate regions in a genome (Figure 1). We tagged virulence and resistance genes of PAIs and REIs through literature search of verified ones. In addition, we adopted known virulence genes from the Virulence Factor Database (VFDB) (13) and resistance genes from the Comprehensive Antibiotic Research Database (CARD) (23) and the Antibacterial Biocide and Metal Resistance Genes Database (BacMet) (24). Transposase genes and integrase genes were excluded from the list. The sequence identifiers of the known virulence and resistance genes (e.g. NCBI accession number) were searched to retrieve amino-acid sequences from GenBank or UniProt website—2266 ea from VFDB, 1833 from CARD and 702 from BacMet. PAI/REI-like regions were identified by checking for the presence of at least one virulence/resistance gene homolog, as described above.

## FUNCTIONALITY UPDATE

### Browse

**Figure 4.**
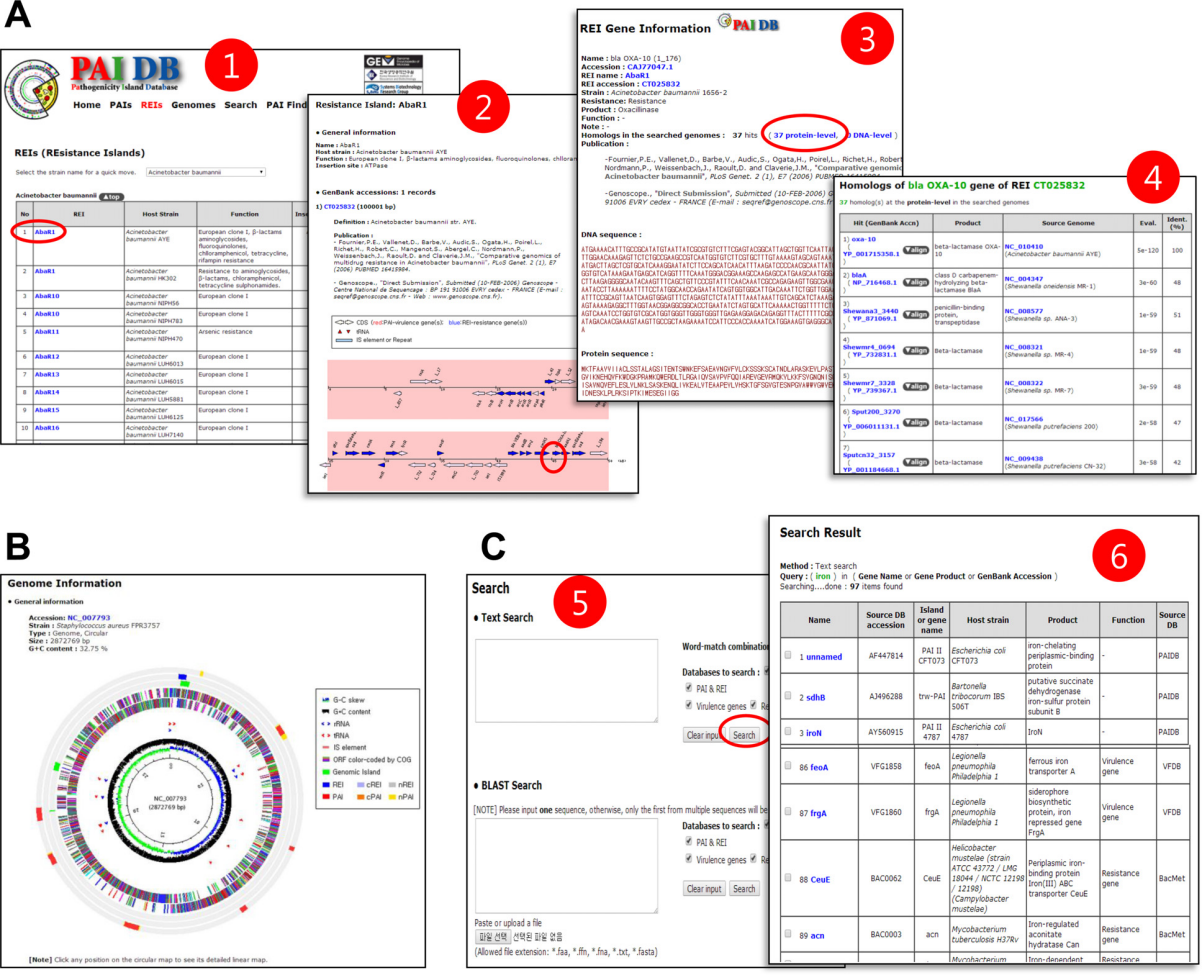
Screenshots of new functional features in PAIDB v2.0. (**A**) Pages for REIs: 1. a list of REIs; 2. information on the clicked REI; 3. information on the clicked gene; 4. homologs of the selected gene in known virulence genes from PAIDB and VFDB, and known resistance genes from PAIDB, CARD and BacMet. (**B**) Circular map showing candidate regions of a selected genome. (**C**) Text and BLAST searches: 5. users can select a database in which to search their text or sequence input; 6. homologs in the BLAST results table are linked to their detailed information (2 and 3). Items clicked on each page that generated the next page are marked in red circles.

PAIDB is freely accessible at http://www.paidb.re.kr. The web-based database was redesigned to offer a user-friendly graphic interface with clear visualization of PAIs, REIs and candidate regions in bacterial genomes. The organization of the website follows the previous version of PAIDB (15). The web pages were modified to reflect the new addition of REI data and to accommodate the significantly expanded content (Figure 4A). The menus ‘PAIs’ and ‘REIs’ enable users to casually explore annotated information on each of PAIs and REIs. The ‘Genomes’ menu provides a list of candidate regions of PAIs and REIs in each microbial genome. When a genome accession number is clicked, the ‘Genome Information’ page shows a circular genome map and tables for PAIs, cPAIs, nPAIs, REIs, cREIs and nREIs (Figure 4B). The circular genome map is clickable and links to a linear genome browser view of the selected genomic region. Each of the candidate regions in table format is linked to the feature table, which contains the genes and virulence/resistance determinants.

### Search tools

The ‘Search’ menu enables users to retrieve PAI and REI data stored in PAIDB through text- and homology-searches (Figure 4C). Along with the PAIDB data, this version of PAIDB allows users to explore information from the databases for virulence factors from PAIDB and VFDB (13) and resistance determinants from PAIDB, CARD (23) and BacMet (24). To facilitate follow-up research, the search results are linked to internal and external databases. The phylogenetic relationship of the selected genes can be inferred through multiple sequence alignment using ClustalW2 (33) .

### PAI finder

In addition to discovering candidate PAI regions in query sequences, ‘PAI Finder’ was modified to also locate candidate REI regions. The overall detection scheme follows Figure 1, except the GI prediction step: BLAT and BLASTX searches against PAIs and REIs, and BLASTX searches against virulence genes and resistance genes. The allowed number of DNA sequences in the multiple FASTA input was increased to 1000 ORFs (approximately 1 Mb). Multithreading, multiprocessing and queuing were implemented to accommodate the volume of the database, the increased number of input sequences and multiple requests by users.

## DISCUSSION

### PAIDB v2.0 allows comprehensive exploration and analysis of PAIs and REIs

Virulence factors and resistance factors are over-represented in large mobile genetic elements of PAIs and REIs present in bacterial pathogens (4,5,34). PAIDB (15) has been a specialized reservoir of all the annotated and candidate PAIs predicted by a method described previously (25). In addition to PAIs, PAIDB v2.0 is now a centralized resource of REIs described so far in the academic literature. The updates included in PAIDB v2.0 are manifold: (i) inclusion of REI data, (ii) improvement of GI detection accuracy, (iii) significantly increased inventory of virulence and resistance genes, (iv) dramatic increase in the number of genomes analyzed and (v) improvement in text- and homology-searches and in the identification system for candidate regions in query sequences.

### Detection of genomic segments homologous to the reported REIs, rather than individual homolog(s), can identify antimicrobial resistance regions in a sequenced genome

GIs are hotspots for the stepwise insertion of different genetic fragments carrying virulence and resistance determinants (5). PAIs often represent mosaic-like structures, such as Hrp PAI in *P. syringae* (35), SPI-2 in *S. typhimurium* (36) and PAI I in verocytotoxin-producing *E. coli* (37). This is also true for REIs, such as SGI1 in *S. typhimurium* (10), PAGI-1 in *P. aeruginosa* (11) and AbaR1 in *A. baumannii* (12). We have previously developed an algorithm that reflects the evolutionary process of PAIs—detection of genomic segments homologous to known PAIs and merging them into a large PAI-like region (25). It should be noted that this approach also reflects disruption and reorganization of a gene cluster during genome reorganization (38) (Figure 3). The algorithm was successfully applied to identify potential PAIs in prokaryotic genomes (15). In this study, we modified and applied the algorithm to identify REIs in prokaryotic genomes, providing 210 cREIs in 178 organisms. As shown in Figure 3, when our method was applied to a genome with primary annotation (39), potential regions related to known PAIs and REIs can be searched and demarcated without human intervention. The predicted region has information regarding the PAIs and REIs constituting it, providing insights into its function and origin.

### The unexpected locations of candidate regions in non-pathogenic organisms allow pathogenomic study of non-pathogenic strains

Virulence factors involved in bacterial pathogenesis are often found in genomes of non-pathogenic bacteria (40,41). Comparative analysis of numerous genome sequences of both pathogenic and non-pathogenic strains of diverse bacterial genera can deepen our understanding of roles of different classes of virulence factors (34,42). In the early version of PAIDB, 171 pathogenic and 108 non-pathogenic prokaryotic genomes derived from 35 classes were analyzed to identify potential PAIs (15). In PAIDB v2.0, the number of genomes analyzed has drastically increased to 1226 pathogenic and 1377 non-pathogenic strains from 90 classes (Figure 2, Supplementary Table S3). While the majority of cPAIs (86%) and cREIs (79%) were detected in pathogenic genomes, they were also found in a small portion of non-pathogenic organisms. The unexpected locations of potential PAIs and REIs in non-pathogenic genomes and their comparison with counterparts in pathogenic genomes may help to clarify the role and mechanism of virulence determinants. Importantly, such analysis may facilitate reassessment of the virulence potential of presumed non-pathogens in light of a better understanding and interpretation of virulence factors.

## CONCLUSION

As the number and diversity of sequenced microbial genomes rapidly accumulate, this web-based, user-friendly resource will continue to contribute to the investigation of genomic regions related to pathogenicity and to give insight into the evolution of pathogenesis. We envision that PAIDB will be of significant use in detecting PAIs and REIs in newly sequenced genomes and mining virulence determinants from metagenomic analyses. Furthermore, as a unique resource for experimentally verified and computationally predicted PAIs and REIs, PAIDB should be particularly useful to design clinical biosensors for pathogen detection and infectious disease diagnostics. PAIDB will continue to incorporate newly discovered PAIs and REIs in a timely manner to keep pace with the rapidly developing field of pathogenomics.

## SUPPLEMENTARY DATA

Supplementary Data are available at NAR Online.
